# Hexa­kis­(dimethyl­formamide-κ*O*)manganese(II) (dimethyl­formamide-κ*O*)pentakis(­thio­cyanato­-κ*N*)chromate(III)

**DOI:** 10.1107/S1600536812023069

**Published:** 2012-05-26

**Authors:** Valentyna V. Semenaka, Oksana V. Nesterova, Vladimir N. Kokozay, Irina V. Omelchenko, Oleg V. Shishkin

**Affiliations:** a Department of Inorganic Chemistry, Taras Shevchenko National University of Kyiv, 64 Volodymyrs’ka St., Kyiv 01601, Ukraine; b STC "Institute for Single Crystals" National Academy of Sciences of Ukraine, 60 Lenina Avenue, Kharkiv 61001, Ukraine

## Abstract

The title compound, [Mn(C_3_H_7_NO)_6_][Cr(NCS)_5_(C_3_H_7_NO)], was obtained unintentionally as a product of an attempted synthesis of heterometallic complexes based on Reineckes anion using manganese powder, Reineckes salt and 1-(2-hy­droxy­eth­yl)tetra­zole as starting materials. The crystal structure of the complex consists of an [Mn(dmf)_6_]^2+^ cation and a [Cr(NCS)_5_(dmf)]^2−^ anion (dmf = dimethyl­formamide). The Mn^II^ and Cr^III^ atoms show a slightly distorted octa­hedral MnO_6_ and CrN_5_O coordination geometries with adjacent angles in the range 85.29 (13)–95.96 (14)°.

## Related literature

For structures including [Mn(dmf)_6_]^2+^ cations, see: Khutornoi *et al.* (2002 ▶); Bencini *et al.* (1992 ▶). For background to direct synthesis, see: Makhankova (2011 ▶).
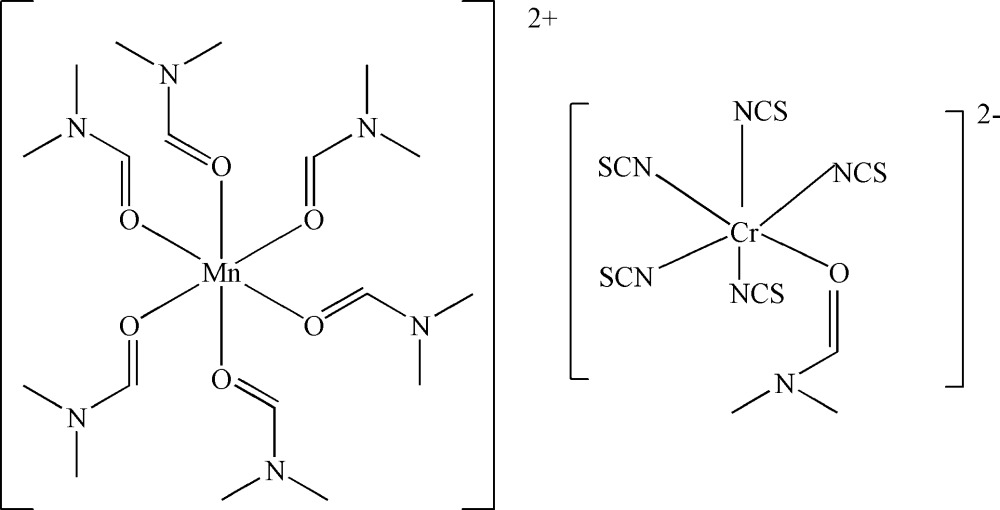


## Experimental

### 

#### Crystal data

[Mn(C_3_H_7_NO)_6_][Cr(NCS)_5_(C_3_H_7_NO)]
*M* *_r_* = 909.01
Monoclinic, 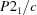

*a* = 15.327 (3) Å
*b* = 17.742 (2) Å
*c* = 17.278 (2) Å
β = 110.36 (2)°
*V* = 4404.9 (11) Å^3^
*Z* = 4
Mo *K*α radiation
μ = 0.82 mm^−1^
*T* = 294 K
0.40 × 0.20 × 0.10 mm

#### Data collection

Oxford Diffraction Xcalibur Sapphire3 diffractometer
Absorption correction: multi-scan (*CrysAlis RED*; Oxford Diffraction, 2010 ▶) *T*_min_ = 0.735, *T*_max_ = 0.922
20206 measured reflections
9620 independent reflections
2863 reflections with *I* > 2σ(*I*)
*R*_int_ = 0.092

#### Refinement

*R*[*F* ^2^ > 2σ(*F* ^2^)] = 0.053
*wR*(*F* ^2^) = 0.100
*S* = 0.67
9620 reflections
463 parameters
4 restraints
H-atom parameters constrained
Δρ_max_ = 0.85 e Å^−3^
Δρ_min_ = −0.57 e Å^−3^

### 

Data collection: *CrysAlis CCD* (Oxford Diffraction, 2010 ▶); cell refinement: *CrysAlis RED* (Oxford Diffraction, 2010 ▶); data reduction: *CrysAlis RED*; program(s) used to solve structure: *SHELXTL* (Sheldrick, 2008 ▶); program(s) used to refine structure: *SHELXTL*; molecular graphics: *XP* in *SHELXTL*; software used to prepare material for publication: *publCIF* (Westrip, 2010 ▶).

## Supplementary Material

Crystal structure: contains datablock(s) I, global. DOI: 10.1107/S1600536812023069/ff2065sup1.cif

Structure factors: contains datablock(s) I. DOI: 10.1107/S1600536812023069/ff2065Isup2.hkl

Supplementary material file. DOI: 10.1107/S1600536812023069/ff2065Isup3.cdx

Additional supplementary materials:  crystallographic information; 3D view; checkCIF report

## Figures and Tables

**Table 1:** Selected bond lengths (Å)

Cr1—N8	1.969 (4)
Cr1—N9	1.977 (4)
Cr1—N11	1.996 (4)
Cr1—O7	1.999 (3)
Cr1—N7	2.002 (4)
Cr1—N12	2.006 (4)
Mn1—O4	2.133 (4)
Mn1—O3	2.140 (4)
Mn1—O1	2.140 (3)
Mn1—O6	2.143 (3)
Mn1—O5	2.167 (3)
Mn1—O2	2.171 (3)

**Fig. 1.**
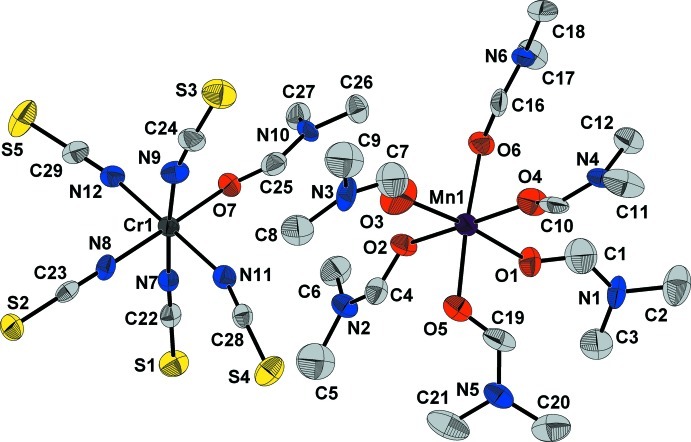
Crystal structure of the complex, showing the atom numbering, with 45% probability displacement ellipsoids

## supplementary crystallographic information

### Comment

Continuing our research on direct synthesis of heterometallic complexes using Reineckes salt, (NH)_4_[Cr(NCS)_4_(NH_3_)_2_]_._H_2_O, as a source of building blocks or metalloligands (Makhankova, 2011), we present here a new Mn^II^/Cr^III^ complex, which was obtained unintentionally as a product of an attempted reaction of manganese powder, Reineckes salt and 1-(2-hydroxyethyl)tetrazole in dmf (dimethylformamide). The crystal structure of the complex consists of a slightly distorted octahedral [Mn(dmf)_6_]^2+^ cation and [Cr(NCS)_5_(dmf)]^+^ anion blocks (Fig. 1). Manganese centers have octahedral coordination environment of six oxygen atoms of dmf ligands. The Mn–O bond lengths vary in the range of 2.133 (4) – 2.171 (3) Å that is in good agreement with those in [Mn(dmf)_6_][Mo_6_Br_8_(NCS)_6_] [2.152 Å (Khutornoi *et al.*, 2002)]. The *cis* and *trans* O–Mn–O bond angles vary from 85.29 (13)° to 95.96 (14)° and from 173.81 (14)° to 175.64 (13)°, respectively. The Cr(III) ions have ON_5_ environment formed by N atoms of NCS groups and O atom of dmf that replace NH_3_ groups of initial complex anion of Reineckes salt. The Cr–N(O) bond lengths vary from 1.969 (4) to 2.006 (4) Å. The *cis* and *trans* N–Cr–N(O) bond angles vary from 86.51 (13)° to 92.83 (16)° and from 175.62 (12)° to 179.24 (17)°, respectively.

### Experimental

Manganese powder (0.069 g, 1.25 mmol), NH_4_[Cr(NCS)_4_(NH_3_)_2_]^.^H_2_O (0.443 g, 1.25 mmol), NH_4_NCS (0.095 g, 1.25 mmol), 1-(2-hydroxyethyl)tetrazole (0.5 g, 2.5 mmol) and dmf (20 ml) were heated to 50–60° and stirred magnetically until total dissolution of the manganese was observed (4.2 h). Dark blue crystals suitable for the X-ray crystallographic study were deposited after successive addition of Pr^i^OH into the resulting blue solution. The crystals were filtered off, washed with dry Pr^i^OH and finally dried *in vacuo* at room temperature. Yield: 0.17 g. Anal. Calc. for C_26_H_49_MnCrN_12_O_7_S_5_: Mn, 6.04; Cr, 5.72; C, 34.35; H, 2.86; N, 18.49; S, 17.63. Found: Mn, 6.0; Cr, 5.9; C, 34.5; H, 3.0; N, 18.6; S, 17.7% IR (KBr, cm^-1^): 3420(w, br), 2964(sh), 2930(w or m), 2807(w), 2116(sh), 2081(*vs*), 1688(sh), 1653(*vs*), 1556(sh), 1496(sh), 1425(sh), 1373(*m*), 1241(m or sh), 1111(*m*), 1058(sh), 971(w), 865(w), 708(sh), 673(*m*), 481(w). The compound is sparingly soluble in dmso and dmf, insoluble in water.

### Refinement

Structure was solved by direct methods and refined against F^2^ within anisotropic approximation for all non-hydrogen atoms. All hydrogen atoms were located geometrically and refined within riding model approximation with C—H = 0.96 (1) Å and *U*_iso_(H)= 1.5Ueq(C) for methyl group H atoms, and C—H = 0.93 (1) Å and *U*_iso_(H)= 1.2Ueq(C) for carbonyl H atoms. O3—C7 bond length was restrained to 1.250 (2) Å value, N3—C7 to 1.330 (3) Å, N3—C8 and N3—C9 to 1.450 (2) Å. Some pairs of atoms (C1 and C7, C3 and C8, C5 and C9) were constrained to have the same anisotropic displacement parameters.

### Crystal data

**Table:**

[Mn(C_3_H_7_NO)_6_][Cr(NCS)_5_(C_3_H_7_NO)]	*F*(000) = 1896
*M**_r_* = 909.01	*D*_x_ = 1.371 Mg m^−^^3^
Monoclinic, *P*2_1_/*c*	Mo *K*α radiation, λ = 0.71073 Å
Hall symbol: -P 2ybc	Cell parameters from 300 reflections
*a* = 15.327 (3) Å	θ = 3.2–28.5°
*b* = 17.742 (2) Å	µ = 0.82 mm^−^^1^
*c* = 17.278 (2) Å	*T* = 294 K
β = 110.36 (2)°	Block, blue
*V* = 4404.9 (11) Å^3^	0.40 × 0.20 × 0.10 mm
*Z* = 4	

### Data collection

**Table:**

Oxford Diffraction Xcalibur Sapphire3 diffractometer	9620 independent reflections
Radiation source: Enhance (Mo) X-ray Source	2863 reflections with *I* > 2σ(*I*)
Graphite monochromator	*R*_int_ = 0.092
Detector resolution: 16.1827 pixels mm^-1^	θ_max_ = 27.5°, θ_min_ = 2.7°
ω scans	*h* = −19→19
Absorption correction: multi-scan (*CrysAlis RED*; Oxford Diffraction, 2010)	*k* = −23→22
*T*_min_ = 0.735, *T*_max_ = 0.922	*l* = −12→22
20206 measured reflections	

### Refinement

**Table:**

Refinement on *F*^2^	Primary atom site location: structure-invariant direct methods
Least-squares matrix: full	Secondary atom site location: difference Fourier map
*R*[*F*^2^ > 2σ(*F*^2^)] = 0.053	Hydrogen site location: inferred from neighbouring sites
*wR*(*F*^2^) = 0.100	H-atom parameters constrained
*S* = 0.67	*w* = 1/[σ^2^(*F*_o_^2^) + (0.0294*P*)^2^] where *P* = (*F*_o_^2^ + 2*F*_c_^2^)/3
9620 reflections	(Δ/σ)_max_ < 0.001
463 parameters	Δρ_max_ = 0.85 e Å^−^^3^
4 restraints	Δρ_min_ = −0.56 e Å^−^^3^
104 constraints	

### Special details

**Table:**

Experimental. Absorption correction: *CrysAlis RED* (Oxford Diffraction, 2010) Empirical absorption correction using spherical harmonics, implemented in SCALE3 ABSPACK scaling algorithm.
Geometry. All e.s.d.'s (except the e.s.d. in the dihedral angle between two l.s. planes) are estimated using the full covariance matrix. The cell e.s.d.'s are taken into account individually in the estimation of e.s.d.'s in distances, angles and torsion angles; correlations between e.s.d.'s in cell parameters are only used when they are defined by crystal symmetry. An approximate (isotropic) treatment of cell e.s.d.'s is used for estimating e.s.d.'s involving l.s. planes.
Refinement. Refinement of *F*^2^ against ALL reflections. The weighted *R*-factor *wR* and goodness of fit *S* are based on *F*^2^, conventional *R*-factors *R* are based on *F*, with *F* set to zero for negative *F*^2^. The threshold expression of *F*^2^ > σ(*F*^2^) is used only for calculating *R*-factors(gt) *etc*. and is not relevant to the choice of reflections for refinement. *R*-factors based on *F*^2^ are statistically about twice as large as those based on *F*, and *R*- factors based on ALL data will be even larger.

### Fractional atomic coordinates and isotropic or equivalent isotropic displacement parameters (Å^2^)

**Table:**

	*x*	*y*	*z*	*U*_iso_*/*U*_eq_	
Cr1	0.22880 (5)	0.21566 (4)	0.23165 (5)	0.0298 (2)	
Mn1	0.70617 (5)	0.21884 (4)	0.26170 (5)	0.0372 (2)	
N1	0.8824 (3)	0.0894 (2)	0.1622 (2)	0.0450 (12)	
N2	0.4621 (3)	0.0800 (2)	0.1678 (3)	0.0393 (11)	
N3	0.6171 (3)	0.3210 (2)	0.4454 (3)	0.0520 (13)	
N4	0.9826 (3)	0.3188 (2)	0.3868 (2)	0.0329 (10)	
N6	0.7430 (3)	0.4134 (2)	0.1329 (3)	0.0361 (10)	
N7	0.1901 (3)	0.1206 (2)	0.1662 (2)	0.0315 (10)	
N8	0.1676 (3)	0.1834 (2)	0.3092 (2)	0.0340 (10)	
N9	0.2747 (3)	0.3103 (2)	0.2927 (3)	0.0423 (12)	
N10	0.3868 (3)	0.3073 (2)	0.1029 (2)	0.0329 (10)	
N11	0.3458 (3)	0.1635 (2)	0.2994 (2)	0.0357 (11)	
N12	0.1107 (3)	0.2669 (2)	0.1626 (2)	0.0353 (11)	
O1	0.7688 (2)	0.14527 (17)	0.1978 (2)	0.0477 (10)	
O2	0.5714 (2)	0.17089 (17)	0.1918 (2)	0.0441 (9)	
O3	0.6391 (3)	0.2817 (2)	0.3308 (3)	0.0773 (13)	
O4	0.8408 (2)	0.2668 (2)	0.3198 (2)	0.0544 (11)	
O5	0.7315 (2)	0.12866 (18)	0.35140 (19)	0.0438 (9)	
O6	0.6900 (2)	0.30489 (18)	0.1707 (2)	0.0447 (9)	
O7	0.2835 (2)	0.24700 (16)	0.14712 (19)	0.0387 (9)	
S1	0.15171 (9)	−0.02018 (7)	0.08949 (9)	0.0478 (4)	
S2	0.06542 (10)	0.13086 (8)	0.40395 (9)	0.0548 (4)	
S3	0.38322 (11)	0.43885 (8)	0.35746 (10)	0.0648 (5)	
S4	0.46635 (9)	0.06510 (8)	0.41462 (9)	0.0527 (4)	
S5	−0.06415 (10)	0.32690 (9)	0.08380 (9)	0.0639 (5)	
C1	0.8415 (5)	0.1480 (4)	0.1847 (4)	0.0816 (15)	
H1A	0.8712	0.1944	0.1908	0.098*	
C2	0.9712 (4)	0.0939 (3)	0.1524 (4)	0.077 (2)	
H2A	0.9950	0.1443	0.1642	0.115*	
H2B	0.9647	0.0811	0.0967	0.115*	
H2C	1.0136	0.0594	0.1898	0.115*	
C3	0.8435 (4)	0.0154 (3)	0.1565 (4)	0.1045 (19)	
H3A	0.7820	0.0187	0.1591	0.157*	
H3B	0.8820	−0.0149	0.2014	0.157*	
H3C	0.8402	−0.0072	0.1051	0.157*	
C4	0.5363 (4)	0.1137 (3)	0.2144 (3)	0.0405 (13)	
H4A	0.5658	0.0952	0.2675	0.049*	
C5	0.4271 (4)	0.0114 (3)	0.1972 (4)	0.0868 (15)	
H5A	0.4554	0.0077	0.2561	0.130*	
H5B	0.4426	−0.0325	0.1720	0.130*	
H5C	0.3608	0.0146	0.1823	0.130*	
C6	0.4090 (4)	0.1043 (3)	0.0861 (3)	0.0569 (16)	
H6A	0.4339	0.1509	0.0747	0.085*	
H6B	0.3454	0.1116	0.0818	0.085*	
H6C	0.4119	0.0668	0.0471	0.085*	
N5	0.8034 (3)	0.0143 (3)	0.3822 (2)	0.0483 (12)	
C7	0.6621 (4)	0.3234 (3)	0.3923 (3)	0.0816 (15)	
H7A	0.7114	0.3568	0.4012	0.098*	
C8	0.5470 (4)	0.2770 (3)	0.4630 (5)	0.1045 (19)	
H8A	0.5281	0.2358	0.4248	0.157*	
H8B	0.4941	0.3083	0.4578	0.157*	
H8C	0.5721	0.2577	0.5183	0.157*	
C9	0.6448 (4)	0.3856 (3)	0.5001 (3)	0.0868 (15)	
H9A	0.6959	0.4108	0.4912	0.130*	
H9B	0.6634	0.3689	0.5564	0.130*	
H9C	0.5933	0.4198	0.4888	0.130*	
C10	0.9062 (4)	0.2806 (3)	0.3830 (3)	0.0426 (14)	
H10A	0.9024	0.2632	0.4325	0.051*	
C11	1.0567 (4)	0.3337 (3)	0.4630 (3)	0.0706 (19)	
H11A	1.0388	0.3172	0.5083	0.106*	
H11B	1.0694	0.3868	0.4679	0.106*	
H11C	1.1116	0.3070	0.4639	0.106*	
C12	0.9917 (4)	0.3480 (3)	0.3130 (3)	0.0528 (15)	
H12A	0.9434	0.3277	0.2659	0.079*	
H12B	1.0513	0.3341	0.3107	0.079*	
H12C	0.9867	0.4020	0.3127	0.079*	
C16	0.7301 (3)	0.3674 (3)	0.1886 (3)	0.0398 (14)	
H16A	0.7518	0.3824	0.2436	0.048*	
C17	0.7130 (3)	0.3912 (3)	0.0463 (3)	0.0555 (16)	
H17A	0.6964	0.3388	0.0413	0.083*	
H17B	0.7628	0.3994	0.0257	0.083*	
H17C	0.6601	0.4208	0.0150	0.083*	
C18	0.7886 (4)	0.4847 (2)	0.1548 (3)	0.0536 (15)	
H18A	0.8060	0.4923	0.2132	0.080*	
H18B	0.7471	0.5242	0.1260	0.080*	
H18C	0.8433	0.4855	0.1397	0.080*	
C19	0.8004 (4)	0.0875 (3)	0.3649 (3)	0.0493 (15)	
H19A	0.8545	0.1096	0.3629	0.059*	
C20	0.8854 (4)	−0.0308 (3)	0.3888 (3)	0.0727 (19)	
H20A	0.9328	0.0012	0.3823	0.109*	
H20B	0.8689	−0.0686	0.3465	0.109*	
H20C	0.9082	−0.0546	0.4420	0.109*	
C21	0.7238 (4)	−0.0250 (3)	0.3849 (3)	0.074 (2)	
H21A	0.6763	0.0106	0.3842	0.110*	
H21B	0.7404	−0.0545	0.4345	0.110*	
H21C	0.7008	−0.0576	0.3378	0.110*	
C22	0.1741 (3)	0.0613 (3)	0.1342 (3)	0.0325 (12)	
C23	0.1256 (3)	0.1623 (2)	0.3479 (3)	0.0297 (12)	
C24	0.3185 (4)	0.3642 (3)	0.3215 (3)	0.0420 (14)	
C25	0.3606 (4)	0.2789 (3)	0.1597 (3)	0.0421 (13)	
H25A	0.4015	0.2822	0.2139	0.050*	
C26	0.4764 (3)	0.3431 (3)	0.1240 (3)	0.0424 (13)	
H26A	0.5055	0.3450	0.1830	0.064*	
H26B	0.4685	0.3935	0.1022	0.064*	
H26C	0.5148	0.3149	0.1009	0.064*	
C27	0.3291 (3)	0.3023 (3)	0.0172 (3)	0.0440 (14)	
H27A	0.2659	0.2927	0.0128	0.066*	
H27B	0.3508	0.2619	−0.0085	0.066*	
H27C	0.3322	0.3489	−0.0100	0.066*	
C28	0.3953 (3)	0.1229 (3)	0.3477 (3)	0.0314 (12)	
C29	0.0390 (4)	0.2920 (3)	0.1304 (3)	0.0383 (13)	

### Atomic displacement parameters (Å^2^)

**Table:**

	*U*^11^	*U*^22^	*U*^33^	*U*^12^	*U*^13^	*U*^23^
Cr1	0.0311 (4)	0.0319 (4)	0.0270 (5)	0.0015 (4)	0.0110 (4)	0.0068 (4)
Mn1	0.0416 (5)	0.0327 (4)	0.0430 (5)	−0.0013 (4)	0.0221 (4)	−0.0004 (5)
N1	0.026 (2)	0.060 (3)	0.051 (3)	0.000 (2)	0.016 (2)	−0.020 (3)
N2	0.043 (3)	0.038 (3)	0.034 (3)	−0.010 (2)	0.011 (2)	−0.001 (2)
N3	0.041 (3)	0.088 (3)	0.036 (3)	0.024 (3)	0.025 (2)	0.012 (3)
N4	0.042 (3)	0.023 (2)	0.028 (3)	−0.009 (2)	0.006 (2)	−0.002 (2)
N6	0.033 (2)	0.029 (2)	0.045 (3)	−0.001 (2)	0.012 (2)	0.011 (2)
N7	0.028 (2)	0.041 (2)	0.026 (3)	0.004 (2)	0.009 (2)	0.009 (2)
N8	0.029 (2)	0.045 (3)	0.028 (3)	0.009 (2)	0.012 (2)	0.001 (2)
N9	0.049 (3)	0.037 (3)	0.037 (3)	0.004 (2)	0.010 (2)	0.006 (2)
N10	0.036 (3)	0.039 (3)	0.030 (3)	−0.002 (2)	0.018 (2)	0.013 (2)
N11	0.038 (3)	0.041 (3)	0.030 (3)	0.001 (2)	0.014 (2)	0.004 (2)
N12	0.042 (3)	0.032 (3)	0.033 (3)	0.002 (2)	0.014 (2)	0.003 (2)
O1	0.043 (2)	0.057 (2)	0.051 (3)	0.001 (2)	0.027 (2)	−0.004 (2)
O2	0.049 (2)	0.0298 (18)	0.055 (3)	−0.0079 (19)	0.021 (2)	0.0002 (19)
O3	0.082 (3)	0.059 (2)	0.083 (3)	−0.003 (2)	0.018 (3)	−0.047 (3)
O4	0.043 (2)	0.055 (3)	0.050 (3)	−0.011 (2)	−0.004 (2)	0.007 (2)
O5	0.047 (2)	0.048 (2)	0.031 (2)	−0.011 (2)	0.007 (2)	0.0022 (19)
O6	0.034 (2)	0.040 (2)	0.053 (3)	−0.0068 (18)	0.0061 (18)	0.0076 (19)
O7	0.040 (2)	0.0407 (19)	0.041 (2)	−0.0003 (18)	0.0208 (18)	0.0063 (17)
S1	0.0461 (9)	0.0402 (8)	0.0479 (10)	−0.0033 (8)	0.0047 (8)	−0.0079 (8)
S2	0.0556 (10)	0.0605 (9)	0.0643 (11)	0.0111 (8)	0.0410 (9)	0.0229 (9)
S3	0.0703 (11)	0.0513 (9)	0.0672 (12)	−0.0134 (9)	0.0169 (10)	−0.0068 (9)
S4	0.0452 (9)	0.0624 (9)	0.0395 (9)	0.0171 (8)	0.0008 (7)	0.0036 (8)
S5	0.0514 (10)	0.0932 (12)	0.0425 (10)	0.0329 (10)	0.0104 (8)	−0.0002 (9)
C1	0.059 (3)	0.084 (4)	0.095 (4)	−0.006 (3)	0.019 (3)	0.003 (3)
C2	0.061 (4)	0.088 (5)	0.088 (5)	−0.001 (4)	0.035 (4)	−0.036 (4)
C3	0.099 (4)	0.053 (3)	0.187 (6)	0.004 (3)	0.083 (4)	0.008 (3)
C4	0.035 (3)	0.043 (3)	0.043 (4)	0.001 (3)	0.014 (3)	−0.008 (3)
C5	0.073 (3)	0.087 (3)	0.082 (4)	−0.012 (3)	0.003 (3)	0.001 (3)
C6	0.054 (4)	0.058 (4)	0.052 (4)	0.004 (3)	0.009 (3)	−0.002 (3)
N5	0.060 (3)	0.051 (3)	0.036 (3)	−0.011 (3)	0.019 (3)	0.009 (3)
C7	0.059 (3)	0.084 (4)	0.095 (4)	−0.006 (3)	0.019 (3)	0.003 (3)
C8	0.099 (4)	0.053 (3)	0.187 (6)	0.004 (3)	0.083 (4)	0.008 (3)
C9	0.073 (3)	0.087 (3)	0.082 (4)	−0.012 (3)	0.003 (3)	0.001 (3)
C10	0.072 (4)	0.024 (3)	0.041 (4)	0.003 (3)	0.030 (3)	0.012 (3)
C11	0.074 (4)	0.061 (4)	0.046 (4)	−0.032 (4)	−0.019 (3)	0.008 (3)
C12	0.056 (4)	0.046 (3)	0.056 (4)	−0.009 (3)	0.019 (3)	0.001 (3)
C16	0.028 (3)	0.055 (4)	0.043 (4)	0.010 (3)	0.022 (3)	0.005 (3)
C17	0.042 (3)	0.068 (4)	0.042 (4)	−0.012 (3)	−0.004 (3)	0.010 (3)
C18	0.061 (4)	0.029 (3)	0.072 (4)	−0.002 (3)	0.024 (3)	0.004 (3)
C19	0.055 (4)	0.041 (4)	0.040 (4)	−0.019 (3)	0.002 (3)	0.008 (3)
C20	0.101 (5)	0.055 (4)	0.060 (5)	0.010 (4)	0.026 (4)	0.009 (4)
C21	0.111 (6)	0.062 (4)	0.042 (4)	−0.035 (4)	0.019 (4)	−0.001 (3)
C22	0.023 (3)	0.048 (3)	0.022 (3)	0.006 (3)	0.002 (2)	0.011 (3)
C23	0.028 (3)	0.029 (3)	0.024 (3)	0.009 (2)	−0.001 (2)	−0.002 (2)
C24	0.054 (4)	0.041 (3)	0.035 (4)	0.003 (3)	0.019 (3)	0.010 (3)
C25	0.041 (3)	0.031 (3)	0.053 (4)	0.003 (3)	0.014 (3)	−0.004 (3)
C26	0.030 (3)	0.055 (3)	0.038 (3)	−0.007 (3)	0.008 (3)	−0.001 (3)
C27	0.035 (3)	0.056 (3)	0.038 (4)	0.006 (3)	0.009 (3)	0.008 (3)
C28	0.031 (3)	0.036 (3)	0.031 (3)	−0.002 (3)	0.015 (3)	−0.005 (3)
C29	0.054 (4)	0.035 (3)	0.029 (3)	0.011 (3)	0.019 (3)	0.002 (3)

### Geometric parameters (Å, º)

**Table:**

Cr1—N8	1.969 (4)	C2—H2C	0.9600
Cr1—N9	1.977 (4)	C3—H3A	0.9600
Cr1—N11	1.996 (4)	C3—H3B	0.9600
Cr1—O7	1.999 (3)	C3—H3C	0.9600
Cr1—N7	2.002 (4)	C4—H4A	0.9300
Cr1—N12	2.006 (4)	C5—H5A	0.9600
Mn1—O4	2.133 (4)	C5—H5B	0.9600
Mn1—O3	2.140 (4)	C5—H5C	0.9600
Mn1—O1	2.140 (3)	C6—H6A	0.9600
Mn1—O6	2.143 (3)	C6—H6B	0.9600
Mn1—O5	2.167 (3)	C6—H6C	0.9600
Mn1—O2	2.171 (3)	N5—C19	1.329 (6)
N1—C1	1.340 (7)	N5—C21	1.421 (6)
N1—C2	1.430 (6)	N5—C20	1.460 (6)
N1—C3	1.432 (6)	C7—H7A	0.9300
N2—C4	1.290 (6)	C8—H8A	0.9600
N2—C6	1.428 (6)	C8—H8B	0.9600
N2—C5	1.488 (6)	C8—H8C	0.9600
N3—C7	1.326 (2)	C9—H9A	0.9600
N3—C8	1.444 (2)	C9—H9B	0.9600
N3—C9	1.451 (2)	C9—H9C	0.9600
N4—C10	1.334 (6)	C10—H10A	0.9300
N4—C12	1.428 (5)	C11—H11A	0.9600
N4—C11	1.434 (6)	C11—H11B	0.9600
N6—C16	1.328 (5)	C11—H11C	0.9600
N6—C18	1.432 (5)	C12—H12A	0.9600
N6—C17	1.457 (5)	C12—H12B	0.9600
N7—C22	1.174 (5)	C12—H12C	0.9600
N8—C23	1.142 (5)	C16—H16A	0.9300
N9—C24	1.174 (5)	C17—H17A	0.9600
N10—C25	1.284 (5)	C17—H17B	0.9600
N10—C27	1.440 (5)	C17—H17C	0.9600
N10—C26	1.441 (5)	C18—H18A	0.9600
N11—C28	1.162 (5)	C18—H18B	0.9600
N12—C29	1.136 (5)	C18—H18C	0.9600
O1—C1	1.213 (6)	C19—H19A	0.9300
O2—C4	1.270 (5)	C20—H20A	0.9600
O3—C7	1.242 (2)	C20—H20B	0.9600
O4—C10	1.223 (6)	C20—H20C	0.9600
O5—C19	1.238 (6)	C21—H21A	0.9600
O6—C16	1.254 (5)	C21—H21B	0.9600
O7—C25	1.260 (5)	C21—H21C	0.9600
S1—C22	1.618 (5)	C25—H25A	0.9300
S2—C23	1.649 (5)	C26—H26A	0.9600
S3—C24	1.642 (6)	C26—H26B	0.9600
S4—C28	1.642 (5)	C26—H26C	0.9600
S5—C29	1.626 (6)	C27—H27A	0.9600
C1—H1A	0.9300	C27—H27B	0.9600
C2—H2A	0.9600	C27—H27C	0.9600
C2—H2B	0.9600		
			
N8—Cr1—N9	92.83 (16)	N2—C6—H6C	109.5
N8—Cr1—N11	90.52 (15)	H6A—C6—H6C	109.5
N9—Cr1—N11	90.03 (16)	H6B—C6—H6C	109.5
N8—Cr1—O7	176.41 (15)	C19—N5—C21	121.6 (5)
N9—Cr1—O7	89.51 (14)	C19—N5—C20	121.0 (5)
N11—Cr1—O7	92.20 (14)	C21—N5—C20	117.0 (5)
N8—Cr1—N7	91.22 (15)	O3—C7—N3	120.8 (5)
N9—Cr1—N7	175.62 (15)	O3—C7—H7A	119.6
N11—Cr1—N7	88.27 (15)	N3—C7—H7A	119.6
O7—Cr1—N7	86.51 (13)	N3—C8—H8A	109.5
N8—Cr1—N12	89.55 (15)	N3—C8—H8B	109.5
N9—Cr1—N12	90.72 (16)	H8A—C8—H8B	109.5
N11—Cr1—N12	179.24 (17)	N3—C8—H8C	109.5
O7—Cr1—N12	87.70 (14)	H8A—C8—H8C	109.5
N7—Cr1—N12	90.97 (15)	H8B—C8—H8C	109.5
O4—Mn1—O3	95.96 (14)	N3—C9—H9A	109.5
O4—Mn1—O1	87.26 (14)	N3—C9—H9B	109.5
O3—Mn1—O1	173.81 (14)	H9A—C9—H9B	109.5
O4—Mn1—O6	84.69 (13)	N3—C9—H9C	109.5
O3—Mn1—O6	94.73 (14)	H9A—C9—H9C	109.5
O1—Mn1—O6	90.83 (12)	H9B—C9—H9C	109.5
O4—Mn1—O5	93.14 (13)	O4—C10—N4	125.3 (5)
O3—Mn1—O5	89.24 (15)	O4—C10—H10A	117.3
O1—Mn1—O5	85.29 (13)	N4—C10—H10A	117.3
O6—Mn1—O5	175.64 (13)	N4—C11—H11A	109.5
O4—Mn1—O2	174.71 (14)	N4—C11—H11B	109.5
O3—Mn1—O2	88.00 (13)	H11A—C11—H11B	109.5
O1—Mn1—O2	89.13 (12)	N4—C11—H11C	109.5
O6—Mn1—O2	91.51 (13)	H11A—C11—H11C	109.5
O5—Mn1—O2	90.41 (12)	H11B—C11—H11C	109.5
C1—N1—C2	123.5 (5)	N4—C12—H12A	109.5
C1—N1—C3	120.3 (4)	N4—C12—H12B	109.5
C2—N1—C3	115.8 (4)	H12A—C12—H12B	109.5
C4—N2—C6	123.5 (4)	N4—C12—H12C	109.5
C4—N2—C5	120.7 (5)	H12A—C12—H12C	109.5
C6—N2—C5	115.8 (4)	H12B—C12—H12C	109.5
C7—N3—C8	140.2 (5)	O6—C16—N6	123.2 (5)
C7—N3—C9	108.7 (4)	O6—C16—H16A	118.4
C8—N3—C9	111.1 (4)	N6—C16—H16A	118.4
C10—N4—C12	119.9 (4)	N6—C17—H17A	109.5
C10—N4—C11	122.7 (4)	N6—C17—H17B	109.5
C12—N4—C11	117.4 (4)	H17A—C17—H17B	109.5
C16—N6—C18	122.2 (5)	N6—C17—H17C	109.5
C16—N6—C17	120.4 (4)	H17A—C17—H17C	109.5
C18—N6—C17	117.4 (4)	H17B—C17—H17C	109.5
C22—N7—Cr1	173.3 (4)	N6—C18—H18A	109.5
C23—N8—Cr1	173.6 (4)	N6—C18—H18B	109.5
C24—N9—Cr1	164.1 (4)	H18A—C18—H18B	109.5
C25—N10—C27	121.2 (4)	N6—C18—H18C	109.5
C25—N10—C26	120.2 (4)	H18A—C18—H18C	109.5
C27—N10—C26	118.5 (4)	H18B—C18—H18C	109.5
C28—N11—Cr1	159.7 (4)	O5—C19—N5	125.4 (5)
C29—N12—Cr1	171.1 (4)	O5—C19—H19A	117.3
C1—O1—Mn1	132.7 (4)	N5—C19—H19A	117.3
C4—O2—Mn1	124.3 (3)	N5—C20—H20A	109.5
C7—O3—Mn1	137.7 (4)	N5—C20—H20B	109.5
C10—O4—Mn1	149.2 (4)	H20A—C20—H20B	109.5
C19—O5—Mn1	120.0 (3)	N5—C20—H20C	109.5
C16—O6—Mn1	121.9 (3)	H20A—C20—H20C	109.5
C25—O7—Cr1	127.3 (3)	H20B—C20—H20C	109.5
O1—C1—N1	125.3 (6)	N5—C21—H21A	109.5
O1—C1—H1A	117.4	N5—C21—H21B	109.5
N1—C1—H1A	117.4	H21A—C21—H21B	109.5
N1—C2—H2A	109.5	N5—C21—H21C	109.5
N1—C2—H2B	109.5	H21A—C21—H21C	109.5
H2A—C2—H2B	109.5	H21B—C21—H21C	109.5
N1—C2—H2C	109.5	N7—C22—S1	179.7 (6)
H2A—C2—H2C	109.5	N8—C23—S2	179.3 (4)
H2B—C2—H2C	109.5	N9—C24—S3	176.3 (5)
N1—C3—H3A	109.5	O7—C25—N10	124.6 (5)
N1—C3—H3B	109.5	O7—C25—H25A	117.7
H3A—C3—H3B	109.5	N10—C25—H25A	117.7
N1—C3—H3C	109.5	N10—C26—H26A	109.5
H3A—C3—H3C	109.5	N10—C26—H26B	109.5
H3B—C3—H3C	109.5	H26A—C26—H26B	109.5
O2—C4—N2	123.6 (5)	N10—C26—H26C	109.5
O2—C4—H4A	118.2	H26A—C26—H26C	109.5
N2—C4—H4A	118.2	H26B—C26—H26C	109.5
N2—C5—H5A	109.5	N10—C27—H27A	109.5
N2—C5—H5B	109.5	N10—C27—H27B	109.5
H5A—C5—H5B	109.5	H27A—C27—H27B	109.5
N2—C5—H5C	109.5	N10—C27—H27C	109.5
H5A—C5—H5C	109.5	H27A—C27—H27C	109.5
H5B—C5—H5C	109.5	H27B—C27—H27C	109.5
N2—C6—H6A	109.5	N11—C28—S4	178.8 (4)
N2—C6—H6B	109.5	N12—C29—S5	179.3 (5)
H6A—C6—H6B	109.5		
			
N8—Cr1—N9—C24	150.7 (15)	O3—Mn1—O6—C16	65.4 (3)
N11—Cr1—N9—C24	60.1 (15)	O1—Mn1—O6—C16	−117.3 (3)
O7—Cr1—N9—C24	−32.1 (15)	O2—Mn1—O6—C16	153.5 (3)
N12—Cr1—N9—C24	−119.7 (15)	N9—Cr1—O7—C25	40.3 (4)
N8—Cr1—N11—C28	17.6 (11)	N11—Cr1—O7—C25	−49.7 (4)
N9—Cr1—N11—C28	110.4 (11)	N7—Cr1—O7—C25	−137.9 (4)
O7—Cr1—N11—C28	−160.1 (11)	N12—Cr1—O7—C25	131.0 (4)
N7—Cr1—N11—C28	−73.6 (11)	Mn1—O1—C1—N1	162.0 (4)
O4—Mn1—O1—C1	−18.3 (5)	C2—N1—C1—O1	−175.8 (6)
O6—Mn1—O1—C1	66.3 (5)	C3—N1—C1—O1	−3.7 (10)
O5—Mn1—O1—C1	−111.7 (5)	Mn1—O2—C4—N2	−170.7 (3)
O2—Mn1—O1—C1	157.8 (5)	C6—N2—C4—O2	−2.0 (7)
O3—Mn1—O2—C4	−85.0 (3)	C5—N2—C4—O2	177.5 (4)
O1—Mn1—O2—C4	89.5 (3)	Mn1—O3—C7—N3	−142.2 (4)
O6—Mn1—O2—C4	−179.7 (3)	C8—N3—C7—O3	8.1 (11)
O5—Mn1—O2—C4	4.2 (3)	C9—N3—C7—O3	−168.0 (5)
O4—Mn1—O3—C7	−10.6 (6)	Mn1—O4—C10—N4	−177.5 (4)
O6—Mn1—O3—C7	−95.8 (6)	C12—N4—C10—O4	1.0 (7)
O5—Mn1—O3—C7	82.5 (6)	C11—N4—C10—O4	179.5 (5)
O2—Mn1—O3—C7	172.9 (6)	Mn1—O6—C16—N6	160.9 (3)
O3—Mn1—O4—C10	55.6 (7)	C18—N6—C16—O6	179.7 (4)
O1—Mn1—O4—C10	−119.1 (7)	C17—N6—C16—O6	−2.2 (6)
O6—Mn1—O4—C10	149.8 (7)	Mn1—O5—C19—N5	−144.6 (4)
O5—Mn1—O4—C10	−34.0 (7)	C21—N5—C19—O5	1.4 (8)
O4—Mn1—O5—C19	−54.4 (4)	C20—N5—C19—O5	173.6 (5)
O3—Mn1—O5—C19	−150.4 (4)	Cr1—O7—C25—N10	−169.8 (3)
O1—Mn1—O5—C19	32.6 (4)	C27—N10—C25—O7	−2.2 (7)
O2—Mn1—O5—C19	121.7 (4)	C26—N10—C25—O7	179.5 (4)
O4—Mn1—O6—C16	−30.2 (3)	Cr1—N12—C29—S5	−77 (43)
